# Aortic pulse wave comparison between controls and triathletes in baseline conditions and in response to acute maximum exercise

**DOI:** 10.1038/s41598-023-43303-7

**Published:** 2023-09-23

**Authors:** Camila Ianê-Siva, Reginaldo Gonçalves, Claudinéia de Oliveira Otoni, Koren C. R. Dias, Ricardo Stein, Bruno Almeida Rezende, Maria Glória Rodrigues-Machado

**Affiliations:** 1 Post-Graduate Program in Health Sciences-MG, Faculdade Ciências Médicas de Minas Gerais (FCM-MG), Belo Horizonte, MG, Brazil; 2 Post-Graduate Program in Sports Sciences, Universidade Federal de Minas Gerais, Belo Horizonte, MG Brazil; 3 Post-Graduate Program in Cardiology and Cardiovascular Sciences, School of Medicine, Hospital de Clínicas de Porto Alegre, Universidade Federal do Rio Grande do Sul, Porto Alegre, Brazil; 4 Exercise Cardiology Research Group (CardioEx), Hospital de Clínicas de Porto Alegre, Universidade Federal do Rio Grande do Sul, Porto Alegre, Brazil

**Keywords:** Physiology, Biomarkers, Cardiology, Health care, Risk factors

## Abstract

To determine the effects of intense training on aortic pulse wave variables and hemodynamic parameters at baseline and at recovery from maximal exercise testing (MaxET) in triathletes compared with sedentary individuals. In this prospective and experimental study, 21 triathletes and 21 sedentary individuals were recruited and evaluated prior and two minutes after the MaxET using the Mobil**-**O-Graph®, which estimates the aortic pulse wave from the brachial artery pressure. The augmentation index (AIx@75) was lower in triathletes after the MaxET compared to control group (16.34 ± 5.95 vs. 23.5 ± 8.53%, p = 0.001), while the pulse wave velocity (PWV) was similar between groups. The heart rate was significantly lower at baseline and after MaxET in triathletes group (55.70 ± 8.95 bpm 91.49 ± 11.39 bpm) compared with control group (62.11 ± 6.70 bpm; 102.08 ± 10.85 bpm). The stroke volume was significantly higher at baseline (96.08 ± 13.96 ml; 86.17 ± 11.24 ml) and after MaxET in triathletes group (69.15 ± 6.51 ml, 58.38 ± 6.99 ml) compared with control group. Triathetes show lower value of AIx@75 after MaxET in comparison with the control group. AIx@75, in addition to being an indirect measure of arterial stiffness, is also a measure of left ventricular afterload. Thus, the lower AIx@75 in triathletes may be due to their lower left ventricular afterload, lower myocardial oxygen demand, and greater coronary perfusion than sedentary individuals. The hemodynamic changes observed in triathletes at rest and during an acute exercise bout are distinctive characteristics of aerobic physical training.

## Introduction

Observational studies have shown inverse associations between physical activity and cardiovascular disease (CVD) in the general population and individuals with high genetic risks^1,2^. Changes in known risk factors of CVD explain part of the beneficial effects of exercise on cardiovascular outcomes. Moreover, exercise directly affects the vascular system through repetitive exposure to forces such as shear stress and transmural pressure, thus promoting structural and functional changes in the vascular wall^3^.

Acute or chronic exercise can modulate various components of the central/aortic pulse wave (APW), composed of forward (ejection) and backward (reflection) waves. The APW analysis allows for the measurement of the arterial stiffness indices, namely the augmentation index (AIx), the central systolic blood pressure (cSBP), and the central pulse pressure (cPP). Similar to the pulse wave velocity (PWV)^4^, the AIx is also associated with cardiovascular risk factors^5^. In response to different stimuli (psychological, mechanical, physical), alterations in blood pressure are associated with variations in the arterial stiffness indices^6^.

The arterial stiffness indices have been evaluated in different exercise modalities, showing a decrease in active individuals, both non-athletes and athletes engaged in team sports^7,8^. In a recent meta-analysis, Ashor et al. observed a significant improvement in PWV and AIx in response to aerobic exercise^9^. The more significant effects were observed in individuals with increased arterial stiffness impairment and participants of longer clinical trials^9^. Chronic exercise has antioxidant, antiatherogenic^10^, and anti-inflammatory effects^11,12^; increases the production of nitric oxide; and reduces the concentration of vasoconstrictor agents^13^. In contrast, it has been proposed that participation in long-term strenuous activity, like marathons and ultramarathons, may not promote additional cardiovascular benefits and may be associated with cardiac alterations and the additional risk of atrial fibrillation^14^.

To fill the gap in this area of knowledge, our goal was to determine the effects of intense training on the APW variables and hemodynamic parameters at baseline, in response to, and at recovery from a maximal exercise testing (MaxET) in triathletes, compared with sedentary individuals.

## Methods

### Study population

Twenty-one triathletes, consisting of young male adults aged between 18 and 45 years who regularly trained for at least 10 h/week, participated in this study. The control group consisted of sedentary volunteers classified as irregularly active, specifically A and B, by the International Physical Activity Questionnaire^15^. This questionnaire asks about the amount of time spent engaging in physical activity during the past week. Questions include activities performed at work, while commuting, for recreation, sport, exercise, or as part of activities at home or in the garden, etc. The classification of volunteers as sedentary or irregularly active, A and B, was based on whether or not they met certain recommendation criteria. A who meets at least one of the criteria for frequency or duration of activity is considered irregularly active. Irregularly active B is one that does not satisfy any of the frequency or duration recommendation criteria. The sedentary volunteer is one who does not engage in any physical activity for at least 10 consecutive minutes per week^16^. Sedentary volunteers were students who were recruited at two universities (one public and one private) from social networks and academic directories. The triathletes were recruited from local clubs and from the state federation of this modality. Interested volunteers who met the inclusion criteria were recruited to participate in the study. They were matched by age, normal blood arterial pressure, and were not on any medication. Volunteers with a smoking history, cardiorespiratory diseases, diabetes, musculoskeletal dysfunctions, and body mass index > 30 kg/m^2^ were excluded.

The experiment was approved by the Research Ethics Committee of the Faculty of Medical Sciences of Minas Gerais, Brazil (CAAE registry number: 97908118.70000.5134).

### Experimental protocol

**Figure 1:**
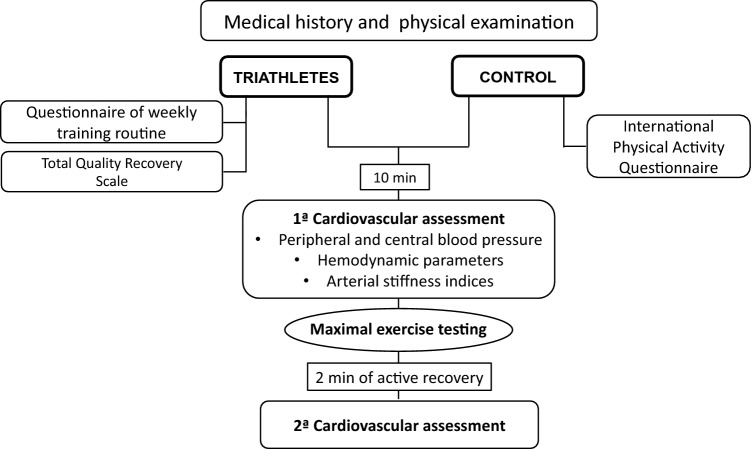
Experimental protocol.

During the experiments, the subjects did not ingest any food or beverage containing caffeine or take any medications. None of the participants exercised 24 h prior to the test. A medical history, physical examination, rest electrocardiogram, and MaxET were obtained to exclude pathologic conditions. All subjects were informed of the risks and discomforts involved in the experiments and gave written informed consent. Subsequently, the triathletes answered a questionnaire regarding their weekly training routine, while the control group answered the International Physical Activity Questionnaire^15^. All the subjects rested in the supine position for 10 min and an assessment of the cardiovascular parameters (Arterial stiffness indices, peripheral and central blood pressure and hemodynamic parameters are also provided (systolic volume, cardiac output, cardiac index and total vascular resistance) was carried out. After 2 min of active recovery following the MaxET, the second assessment of the cardiovascular parameters was performed (Fig. 1).

### Skinfold thickness

The skinfold thickness was measured using a clinical caliper (Saehan Skinfold Caliper—Korea) with a 1-mm precision at the pectoralis, midaxilla, triceps, subscapula, suprailiac, and thigh^17^.

### Assessment of aerobic functional capacity

MaxET was carried out using the Balke protocol, with a graded exercise test and without intervals^18^. To do so, the triathletes’ bicycles were attached to a simulator (Computrainer, Racer Mate—USA), properly calibrated according to the manufacturer's instructions. Meanwhile, the control group’s assessment was carried out using a Criterium bike (Sense bikes—Brazil), attached to the same simulator. A 5-min warm-up period was provided. The test was initiated at an intensity of 50 watts for both groups, with an auto-selected cadence between 80 and 100 rpm. The power was increased by 50 watts after 2 min for the athletes and 25 watts for the sedentary group. All tests were carried out until exhaustion, as determined by the volunteer or when the intensity could not be upheld. The Borg scale was used to assess the volunteers during the MaxET^19^.

In addition to the hemodynamic variables, the maximum heart rate (HRmax), power (watts), and effort time were evaluated. The maximal oxygen uptake (VO_2_max), in relation to body mass, was estimated using Balke’s equation: [(watts by the end of the test × 12) + 200/body mass in kg)]^18^.

The athletes’ recovery from their last training session was assessed using the Total Quality Recovery Scale, on a scale of 6 to 20, with 6 representing no recovery and 20 representing full recovery.

### Cardiovascular assessment

**Figure 2:**
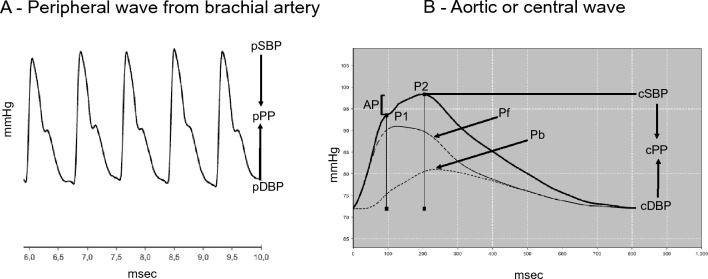
Pulse waves from the brachial (**A**) and aortic (**B**) arteries. P1 refers to the first systolic peak caused by the contraction of the left ventricle, while P2 represents to the second systolic peak caused by reflection wave. The difference between these peaks represents the central augmentation pressure (cAP = P2–P1), indicating the degree in which the systolic blood pressure (cSBP) increases according to the reflection wave. The ratio of the cAP and cPP represents the augmentation index (AIx), which is corrected for a heart rate of 75 bpm (AIx@75). *Pf* Forward or ejection wave, *Pb* Backward or reflection wave, *cDBP* Central diastolic blood pressure, *cPP* central pulse pressure, difference between cSBP and cDBP.

The components of the APW and hemodynamic parameters were assessed using the non-invasive Mobil-O-Graph® (IEM, Germany), which utilizes the oscillometric method of blood pressure measurement from the brachial artery^20–22^. The APW (Fig. 2) is the sum of the incident (forward wave, Pf) and reflected (backward wave, Pb) waves. The AIx is characterized by two peaks of pressure^4^, with the first (P1) one resulting from the contraction of the left ventricle and the second (P2) resulting from the reflection wave. The difference between these peaks represents the central augmentation pressure (cAP = P2–P1), indicating the degree to which the systolic blood pressure increases according to the reflection wave. The ratio between the cAP or P2–P1 and cPP defines the AIx [AIx = (P2 − P1)/cPP × 100]. Given the strong influence of HR, the AIx was adjusted to the HR of 75 bpm. The PWV was estimated by a mathematical model, considering several parameters obtained in the analysis of the APW and the wave separation^20^. Previous validation studies showed acceptable agreement between Mobil-O-Graphy derived parameters and invasive^23^ and noninvasive^24^ measurements.

The basal measure was done with the patient lying on a stretcher after 10 min of rest. After the conclusion of the MaxET, a 2-min active recovery period with the same intensity as the beginning of the test was provided, followed by a new measurement of the cardiovascular parameters.

The device performed three consecutive measurements and the mean of the three was considered for the final analysis^21^.

### Sample calculation

Twenty-one triathletes and 21 controls were needed to detect a minimum difference of 3.5 between the AIx@75 values of both groups at a 5% significance level and 80% minimum power. This study utilized the AIx@75 standard deviation from a previous study^25^.

### Statistical analysis

The qualitative variables are presented as relative and absolute frequencies, while the quantitative variables are presented as mean ± standard deviation. The quantitative variables were submitted to the Shapiro–Wilk normality test.

Two-way analyses of variance models for repeated measures were constructed to assess the differences between (sedentary vs. triathletes) and within the groups (pre and post-exercise). For post-hoc comparisons, the paired samples Student's t-test and Student's t-test for independent samples between groups were utilized, with p-values adjusted with Bonferroni corrections. To assess the effects of anthropometric characteristics, maximal aerobic power, and VO_2_max related to the AIx@75, and PWV, models of multiple linear regression were created for both groups. The final model was produced by including the independent variables with p < 0.20 in the association analysis with the AIx@75 and PWV using an applied stepwise strategy using a saturated model. In the said model, the age variable was retained, regardless of its significance. The quality of the adjustment was assessed by the adjusted R2 and the residual analysis. The analyses were performed in the free software R 3.5.1 using a significance level of 5%.

### Ethics approval

This study was performed in line with the principles of the Declaration of Helsinki. Approval was granted by the Research Ethics Committee of the Faculty of Medical Sciences of Minas Gerais (Registry number: 97908118.70000.5134).

### Consent to participate

Written informed consent was obtained from all the participants.

## Results

Twenty-one triathletes and 21 sedentary or irregularly active volunteers, who formed the control group, participated in this study. Regarding the level of physical activity of the control group, 33.3% of the participants were classified as irregularly active A, 52.4% were classified as irregularly active B, and 14.3% were considered sedentary. The athletes, who trained for a weekly total of 17.43 ± 5.09 h, scored 15.67 ± 1.44 on the Total Quality Recovery, indicating that they were “well recovered” from their last training session.

Table 1 shows the anthropometric data (Age, height, weight, body mass index and body fat) and the MaxET-related variables of the control and triathletes groups. The weight and body fat percentage were significantly lower in the triathletes group. Compared to the control group, at MaxET, VO_2_max was significantly higher, and resting HR was significantly lower in the triathletes group.

**Table 1:** Anthropometric and maximal exercise test data.

Parameters	Controls	Triathletes	p-value
Age (years)	27.90 ± 5.12	32.00 ± 8.30	0.063
Height (m)	1.77 ± 0.06	1.75 ± 0.05	0.190
Weight (kg)	79.82 ± 14.01	72.60 ± 5.35	0.037
BMI (kg/m^2^)	25.32 ± 3.77	23.70 ± 1.76	0.084
Body fat (%)	15.35 ± 5.71	7.13 ± 2.30	< 0.0001
Maximum aerobic power (W)	193.31 ± 35.35	333.17 ± 22.97	< 0.0001
Relative maximum aerobic power (W/kg)	2.46 ± 0.57	4.65 ± 0.49	< 0.0001
Resting heart rate (bpm)	62.86 ± 6.73	54.71 ± 6.72	0.0033
HRmax (bpm)	183.90 ± 12.85	180.57 ± 12.02	0.5025
VO_2_max (ml/kg/min)	32.09 ± 7.16	58.28 ± 5.85	< 0.0001

The data are represented as mean ± SD. The p-values refer to the Student's t-test for independent samples. *BMI* body mass index, *HRmax* maximum heart rate, *VO*_*2*_*max* maximal oxygen uptake.

Regarding the pre-exercise baseline assessment, no differences in the central and peripheral arterial pressures were observed between groups (p > 0.05) (Table 2). The HR was significantly lower (55.70 ± 8.95 bpm, 62.11 ± 6.70 bpm; p = 0.012), and the SV was significantly higher (96.08 ± 13.96 ml, 86.17 ± 11.24 ml; p = 0.015) in the athletes compared to control group. TVR and cardiac output did not differ between groups (p > 0.05) (Fig. 2).

**Table 2:** Cardiovascular parameters assessed in basal conditions (pre) and immediately after (post) MaxET between the groups (controls and triathletes).

Parameters	Controls	Triathletes	p-value* between groups*	p-value* intra groups*
pSBP (mmHg)				
Pre-exercise	125.11 ± 8.58	118.79 ± 6.93	0.714	0.026
Post-exercise	124.60 ± 9.48	128.52 ± 10.62*		
pDBP (mmHg)				
Pre-exercise	75.51 ± 8.07	71.21 ± 6.56	0.342	0.008
Post-exercise	67.03 ± 9.90*	69.67 ± 8.49		
pMAP (mmHg)				
Pre-exercise	98.24 ± 6.78	93.02 ± 6.05	0.687	0.685
Post-exercise	93.41 ± 7.87	96.52 ± 7.55		
pPP (mmHg)				
Pre-exercise	49.60 ± 9.68	47.59 ± 6.23	0.246	< 0.001
Post-exercise	57.57 ± 11.41*	58.86 ± 11.66*		
cSBP (mmHg)				
Pre-exercise	115.79 ± 7.76	109.13 ± 6.77	0.564	0.116
Post-exercise	112.94 ± 6.35	117.98 ± 10.94		
cDBP (mmHg)				
Pre-exercise	76.43 ± 8.09	72.37 ± 6.61	0.370	0.028
Post-exercise	68.98 ± 9.78*	71.59 ± 8.33		
cPP (mmHg)				
Pre-exercise	39.46 ± 8.68	36.81 ± 6.38	0.134	< 0.001
Post-exercise	43.92 ± 7.80	46.43 ± 10.22*		

The data are represented as mean ± SD. *pSBP* peripheral systolic blood pressure, *pDBP* peripheral diastolic blood pressure, *pMAP* peripheral mean arterial pressure, *pPP* peripheral pulse pressure, *cSBP* central systolic blood pressure, *cDBP* central diastolic blood pressure, *cPP* central pulse pressure. *p < 0.05 in comparison with pre-exercise.

In the triathletes group, the amplitude of both waves increased after the MaxET (Table 3). However, the other parameters of arterial stiffness were similar in both groups in baseline conditions.

**Table 3:** Arterial stiffness indexes assessed in basal conditions (pre) and immediately after (post) exercise between the groups (controls and triathletes).

Parameters	Controls	Triathletes	p-value* between groups*	p-value* intra groups*
Augmentation pressure (mmHg)			0.612	0.115
Pre-exercise	6.25 ± 3.57	6.73 ± 3.86		
Post-exercise	5.81 ± 3.33	4.83 ± 2.79		
Reflection coefficient (%)			0.446	0.733
Pre-exercise	65.03 ± 4.58	62.90 ± 8.00		
Post-exercise	66.05 ± 8.29	64.27 ± 7.26		
Ejection wave amplitude (mmHg)			0.392	< 0.01
P re-exercise	25.47 ± 5.33	24.09 ± 3.61		
Post-exercise	27.99 ± 4.32	29.71 ± 5.87*		
Reflection wave amplitude (mmHg)			0.252	< 0.01
Pre-exercise	16.56 ± 3.69	15.27 ± 3.19		
Post-exercise	18.61 ± 4.18	19.18 ± 4.63*		
AIx@75 (%)			0.118	0.001
Pre-exercise	11.95 ± 8.57	13.43 ± 11.92		
Post-exercise	23.5 ± 8.53*	16.34 ± 5.95^#^		
PWV (m/s)			0.420	0.060
Pre-exercise	5.46 ± 0.42	5.50 ± 0.51		
Post-exercise	5.46 ± 0.39	5.92 ± 0.62		

The data are represented as mean ± SD. *AIx@75* Augmentation index normalized by heart rate of 75 bpm. PWV: Pulse wave velocity. *p < 0.05 in comparison with pre-exercise. ^‡^p < 0.05 in comparison with the control group.

After MaxET, the peripheral diastolic blood pressure (pDBP) and central DBP (cDBP) decreased, while the peripheral pulse pressure (pPP) increased significantly in the control group. Meanwhile, in the triathletes group, the peripheral SBP (pSBP), pPP, and central peripheral pulse (cPP) increased significantly after MaxET (Table 2).

**Figure 3:**
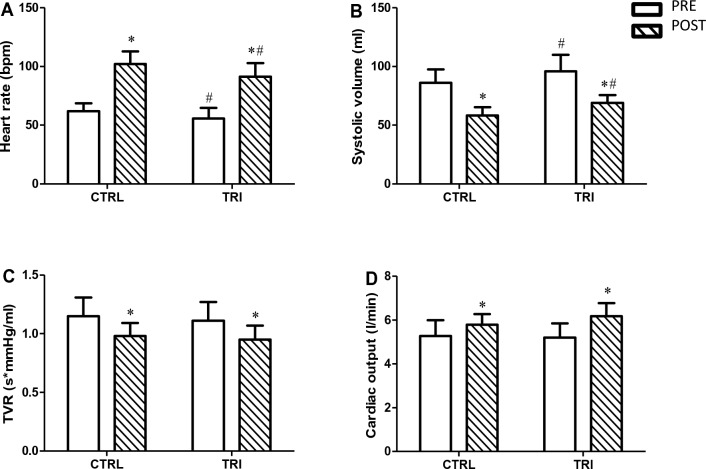
Hemodynamic parameters assessed in basal conditions (pre) and immediately after (post) exercise between control (CTRL) and triathletes groups. (**A**) Heart rate, (**B**) Systolic volume, (**C**) Total vascular resistance, (**D**) Cardiac output. *p < 0.05 in comparison with pre-exercise. ^#^p < 0.05 in comparison with the control group at the same time point.

The MaxET increased the HR significantly in both groups, but it was significantly lower in triathletes than in the control group. Though SV decreased significantly after the MaxET in both groups, it was significantly higher in the triathletes group. Furthermore, TVR decreased significantly in both groups after the MaxET (Fig. 3).

Regarding the stiffness parameters, the MaxET did not alter the augmentation pressure and the PWV (Table 3). In contrast, the ejection and reflection waves’ magnitudes were significantly higher in the triathletes group after MaxET.

The associations between the PWV and AIx@75 were investigated using linear regression models. In the control group, AIx@75 correlated negatively with SV (P < 0.01, r = − 0.69) but positively with PPp (p = 0.01, r = 0.55). In the triathletes group, AIx@75 correlated negatively with SV (p = 0.01, r = − 0.54). In the control group, PWV was positively correlated with age (p < 0.01, r = 0.72), pSBP (p  <  0.01, r = 0.64), pDBP (p < 0.01, r = 0, 77), mean arterial pressure (pMAP; p < 0.01, r = 0.87), cSBP (p < 0.01, r = 0.87), cDBP (p  < 0.01, r = 0.76), SV (p  = 0.03, r = 0.47), and TVR (p < 0.01, r = 0.56), but was negatively correlated with HR (p = 0.03, r = − 0.48). In the triathletes group, PWV was positively correlated with age (P < 0.01, r = 0.73), pSBP (p < 0.01, r = 0.56), pMAP (p  < 0.01, r = 0.57), and cSBP (p  < 0.01, r = 0.68), but was negatively correlated with pulse pressure amplifications (PPA).

Table 4 shows the multiple linear regression equations for AIx@75% and PWV as dependent variables in both groups.

**Table 4:** Linear regression model for both groups.

Variables	Equation	Adjusted R^2^
AIx@75 controls	19.997 − Age (0.219) + pSBP (0.602) − SV (1.076)	81.30%
AIx@75 triathletes	40.149 + Age (0.171) + pPP (0.308) − SV (0.654)	49.32%
PWV controls	0.621 + Age (0.040) + pDBP (0.040) + cPP (0.047) − RC (0.015)	96.49%
PWV triathletes	0.628 + Age (0.057) + cDBP (0.022) + cPP (0.041)	95.18%

All models are presented normal, homoscedastic residues without outliers. *AIx@75* Augmentation index corrected to 75 bpm heart rate, *PWV* Pulse wave velocity, *pSBP* Peripheral systolic blood pressure, *SV* Systolic volume, *PP* Pulse pressure, *pDBP* Peripheral diastolic blood pressure, *cPP* Central pulse pressure, *cDBP* Central diastolic blood pressure, *RC* Reflection coefficient.

## Discussion

As far as we are aware, this is the first study that evaluated and compared the effects of training on the APW variables and hemodynamic parameters, at baseline, in response to, and during recovery of a MaxET between the triathletes and sedentary individuals. The arterial stiffness indices were assessed through the PWV, the gold standard in terms of arterial stiffness assessment, and the AIx@75, a measurement reflecting arterial stiffness and the peripheral waves. Although the arterial stiffness indices are associated with low levels of physical activity and sedentary behavior, in this study, the PWV did not differ between triathletes and the control group in baseline conditions. Controversy exists regarding the acute effects of intensive exercise and recovery on arterial stiffness. In the present study, PWV did not change with exercise in both control and triathlete groups. Similarly, a recent study found that acute aerobic exercise (30 min of cycling at 70–75% of HRmax) did not alter the PWV values, assessed before and at 10-min intervals for 60 min after the intervention^26^. On the other hand, Muller et al. observed that PWV increased during exercise in comparison to baseline and thirty minutes after terminating the workout, a drop in PWV below baseline was observed^27^. Therefore, the authors found that moderate or vigorous exercise temporarily increases arterial stiffness in middle- and long-distance athletes. Meanwhile, analyzing the chronic adaptations of these indices in marathon runners, Vlachopoulus et al. observed that the PWV was significantly higher in marathon runners than in the control group, but the AIx@75 was similar in both groups^4^. The difference in these results may be related to the association of the three aerobic modalities in triathlons compared to the unique long-distance modality in marathoners. In the current study, predictors of PWV increase in the control group were age, pDBP, cPP, and refection coefficient (R2 = 96.49%), while age, cDBP, and cPP (R2 = 95.18%) were noted in the triathletes group.

Under baseline conditions, AIx@75 did not differ between the controls and triathletes. On the other hand, after the MaxET, the AIx@75 increased significantly in the control group and did not change in triathletes. Considering only post-exercise period triathletes group presented AIx@75 significantly lower compared to control group. Many factors determine AIx@75 since it can be positively influenced by the rapid return^28^ and magnitude of the reflection wave^29^, while it is negatively associated with the heart rate^30^. The forward wave is generated primarily by ventricular contraction, and its amplitude is mainly determined by ventricular contraction and the PWV of the proximal aorta^31^. On the other hand, the backward wave is influenced by the characteristics of the peripheral vasculature at the major sites of reflection. In the present study, the amplitude of the ejection and reflection waves did not change in the control group after a MaxET. In contrast, the amplitude of both waves increased in the triathletes group after the MaxET. Lower levels of AIx@75 in the triathletes, possibly due to the positive effects of training on cardiovascular performance, are associated with lower ventricular afterload and increased myocardial oxygen demand and coronary perfusion^32^. Exercise intensity affects pulse wave reflection, with different time courses for AIx@75 post-exercise. Hanssen et al. investigated the acute and 24-h effects of high-intensity interval training and moderate continuous training on arterial pulse wave reflection before and 5 (t5), 20 (t20), 35 (t35), and 50 (t50) minutes after the acute exercise bouts^33^. The AIx@75 increased after both acute exercise types but was higher after high-intensity interval training at t5, t20 and t35 compared to moderate continuous training. The 24-h follow-up revealed a signficant decline in AIx@75 after high-intensity interval training but not after moderate continuous training, indicating more favourable effects on pulse wave re!ection compared to moderate continuous training. This may result in substantial positive chronic training effects on arterial stiffness in health and cardiovascular disease.

Hemodynamically, though the cardiac indices were similar in both groups, the triathletes group presented with higher SV and lower resting HR than the sedentary group. The decrease in HR and increase in SV are responses to aerobic physical training, such as running and swimming, leading to eccentric ventricular hypertrophy due to an increase in the left ventricle’s internal diameter^34^. Morphological adaptations associated with chronic aerobic physical activity, referred to as aerobic exercise training-induced cardiac remodeling, lead to increased cardiac output during exercise, promoting a significant increase in the maximal VO_2_ after training^35^.

The pPP also increased significantly in both groups after MaxET. This augmentation in pPP can occur by increasing the pSBP, reducing the pDBP, or a combination of the two. In the control group, this occurred due to the significant reduction in pDBP, while the significant increase in pSBP was the determining factor for the significant increase in pPP in triathletes. Of interest, the cPP did not change in the control group after exercise, despite the significant reduction in cDBP. In the trained group, changes in the cSBP and cDBP significantly increased the cPP. Although the maximum aerobic power achieved by triathletes was 58% higher than the control group, this variable did not correlate with the cPP and cPP. In the present study, the final MaxET power in the control group was negatively correlated with age, pDBP, pMAP, cDBP, and TVR, and positively with pPP and cPP. In triathletes, the final MaxET power correlated positively with the pSBP, cSBP, and pMAP.

Regarding the hemodynamic parameters, the HR and cardiac output increased significantly after the MaxET in both groups, as expected. Moreover, the HR was lower among the triathletes, while the SV and cardiac output were significantly higher after the MaxET. The post-exercise increase in cardiac output was determined by the increase in the HR, since the SV decreased, probably due to the reduction in the TVR and consequently in the venous return (pre-load). Similar results were found by Karabulut et al.^36^. These authors evaluated the effects of the duration of moderate-intensity treadmill exercise (65% of VO_2_max at 30, 45, and 60 min of exercise) in physically active, healthy individuals but did not perform structured, regular physical training regularly. Their findings verified that the treadmill exercise resulted in increased HR and reduced TVR and SV. Pierce et al. assessed the impact of the type of exercise (acute aerobic exercise—30 min of cycling at 70–75% of HRmax vs. resistance exercise) among healthy adult males (26.7 ± 7.2 years old), with a minimum experience of 3 months in aerobic and resistance exercises^26^. Upon measuring and analyzing the cardiovascular parameters in 10-min intervals for 60 min, they observed that, after both exercises, the HR was higher at all stages of the assessment compared to the control group. In addition, at most of the measurement intervals, the HR values were significantly higher after the resistance exercise compared to the acute aerobic exercise.

To the best of our knowledge, this was the first study that assessed the acute response and chronic adaptations on arterial stiffness indexes and vascular and hemodynamic parameters in triathletes. However, our study had some limitations. This study only had male volunteers, making it impossible to generalize the data for female athletes. Many studies have shown that there are sex-related differences in the progression of arterial stiffness associated with aging and the risk factors of CVD^37^.

## Conclusion

The lower value of AIx@75 observed in triathletes after MaxET indicates that they are exposed to lower left ventricular afterload, lower myocardial oxygen demand, and greater coronary perfusion than are sedentary individuals. The hemodynamic changes (lower HR and higher SV) observed in triathletes at rest and during an acute exercise bout are outstanding effects of aerobic physical training.

## Data Availability

The datasets analysed during the current study is available from the corresponding author on reasonable request.

## Abbreviations

AIx: Augmentation index
AIx@75%: AIx adjusted to the HR of 75 bpm
APW: Central/aortic pulse wave
CVD: Cardiovascular disease
DBP: Diastolic blood pressure
HR: Heart rate
HRmax: Maximum heart rate
MAP: Mean arterial pressure
MaxET: Maximal exercise training
Pb: Backward wave
Pf: Forward wave
P1: First systolic peak caused by the contraction of the left ventricle
P2: Second systolic peak caused by reflection wave
PP: Pulse pressure
PWV: Pulse wave velocity
SBP: Systolic blood pressure
SV: Systolic volume
VO_2_max: Maximal oxygen uptake
